# Introduction

**DOI:** 10.1155/S146392469300001X

**Published:** 1993

**Authors:** Peter B. Stockwell


					Journal of Automatic Chemistry, Vol. 15, No. 1 (January-February 1993), p. 1
Special Issue: International Symposium on
Laboratory Automation and Robotics 1992.
Management Papers
Volume 15, Number 1, is devoted to papers presented at the tenth International Symposium on
Laboratory Automation and Robotics, which was held in Boston, USA from 25 to 28 October 1992.
The issue includes selected papers on Managing Automation and Robotics, the keynote addresses
are by Eugene McGonigle and Sein O'Connor and the closing address by Frank Zenie.
A full report on the meeting was published inJournal ofAutomatic Chemistry, Vol. 14, No. 6, together
with the abstracts of all tle papers and posters presented at the Symposium.
The papers in this issue are reprinted, with permission, from the Proceedings ofthe 1992 International
Symposium on Laboratory Automation and Robotics. I would like to thank all those at the Zymark
Corporation, especially Sharon Correia, Zymark Corporation, Zymark Center, Hopkinton,
Massachusetts 01748, USA (Tel: 508 435 9500; Fax 508 435 3439).
CONTENTS
Practical aspects of laboratory automation in pharmaceutical development, E.J. McGonigle, p. 3.
Rational drug design, medicinal chemistry, planned serendipity and the impact ofautomation on the
drug discovery process, S. O'Connor, p. 9.
Laboratory automation: changing the role of the technical professional, K.J. Halloran, p. 13.
Managing the design and implementation ofan agrochemical central sample preparation laboratory,
A. N. Mannherz, p. 19.
Superior productivity- in the laboratory and beyond, F. H. Zenie, p. 23.
PETER B. STOCKWELL

				

